# Real-World Data-Derived Pharmacovigilance on Drug-Induced Cognitive Impairment Utilizing a Nationwide Spontaneous Adverse Reporting System

**DOI:** 10.3390/medicina60071028

**Published:** 2024-06-23

**Authors:** Yongjun Sunwoo, Sae Hyun Eom, Ji Seong Yun, Yujin Kim, Jeongmin Lee, Soo Hyeon Lee, Sooyoung Shin, Yeo Jin Choi

**Affiliations:** 1 Department of Regulatory Science, Graduate School, Kyung Hee University, Seoul 02447, Republic of Korea; 2 Institute of Regulatory Innovation through Science (IRIS), Kyung Hee University, Seoul 02447, Republic of Korea; 3 Department of Pharmacy, College of Pharmacy, Kyung Hee University, Seoul 02447, Republic of Koreayjs39@khu.ac.kr (J.S.Y.);; 4 Department of Pharmacy, College of Pharmacy, Ajou University, Suwon 16499, Republic of Korea; 5 Research Institute of Pharmaceutical Science and Technology (RIPST), Ajou University, Suwon 16499, Republic of Korea

**Keywords:** pharmacovigilance, cognitive impairment, cancer, chemotherapy, Parkinson’s disease, real-world data

## Abstract

*Background and Objectives*: Despite high incidences of cognitive impairment with aging, evidence on the prevalence and the seriousness of drug-induced cognitive impairment is limited. This study aims to evaluate the prevalence and the severity of drug-induced cognitive impairment and to investigate the clinical predictors of increased hospitalization risk from serious drug-induced cognitive impairment. *Materials and Methods*: Adverse drug events (ADEs) regarding drug-induced cognitive impairment reported to the Korean Adverse Event Reporting System Database (KAERS DB) from January 2012 to December 2021 were included (KIDS KAERS DB 2212A0073). The association between the etiologic classes and the reporting serious adverse events (SAEs) was evaluated using disproportionality analysis, and the effect was estimated with reporting odds ratio (ROR). Clinical predictors associated with increased risk of hospitalization from SAEs were identified via multivariate logistic analysis, and the effect was estimated with odds ratio (OR). *Results*: The most etiologic medication class for drug-induced cognitive impairment ADEs was analgesics, followed by sedative-hypnotics. Anticancer (ROR 57.105, 95% CI 15.174–214.909) and anti-Parkinson agents (ROR 4.057, 95% CI 1.121–14.688) were more likely to report serious drug-induced cognitive impairments. Male sex (OR 19.540, 95% CI 2.440–156.647) and cancer diagnosis (OR 18.115, 95% CI 3.246–101.101) are the major clinical predictors for increased risk of hospitalizations due to serious drug-induced cognitive impairment. *Conclusions*: This study highlights the significant prevalence and severity of drug-induced cognitive impairment with cancer diagnosis and anticancer agents. However, further large-scaled studies are required because of the potential underreporting of drug-induced cognitive impairments in real practice settings, which is further contributed to by the complexity of multiple contributing factors such as comorbidities.

## 1. Introduction

Cognitive impairment refers to a broad spectrum of dysfunction in crucial cognitive functions, including memory, learning, perception, and problem solving, which are essential for daily life and complex decision making [1]. Cognitive impairment is considered a major symptom of various neurological disorders, especially Alzheimer’s disease, manifesting as substantial decline in memory, language, and cognitive clarity [2]. Moreover, it is also observed in patients with psychiatric disorders such as schizophrenia, major depressive disorders, and bipolar disorders, where prominent cognitive deficits severely impact quality of life and daily functioning [3]. Cognitive impairment has profound implications as it affects individual autonomy, employment capabilities, and social interactions [1]. Moreover, cognitive impairment is closely correlated with substantial economic impact, as evidenced by substantial increased healthcare costs [4]. Studies suggest there will be a significant increased global burden with dementia, estimated to exceed USD 16.9 trillion by 2050 [5]. However, evidence regarding optimal management strategies as well as the economic burden of cognitive impairment, aside from dementia, is currently limited despite its substantial societal and economic effects [6]. Moreover, as age is a crucial risk factor for cognitive impairment associated with various medical conditions, the increasing number of aging populations will markedly elevate the incidence of cognitive impairment in the elderly, indicating the importance of comprehensive research on the management and etiologies of cognitive impairment to ensure optimal patient prognoses [7].

Cognitive impairment can also arise as an adverse drug event (ADE) of pharmacological interventions [8]. Pharmacological agents involving psychoactive drugs, antidepressants, anticholinergics, benzodiazepines, and anticonvulsants are associated with increased risk of drug-induced cognitive impairment [9]. Notably, anticholinergics have been identified as a considerable risk factor for drug-induced cognitive impairment, manifested as confusion and memory impairment, especially in the elderly [9]. Moreover, a French population-based cohort study revealed the markedly elevated risk of dementia in continuous anticholinergic users, implying a considerable impact of drug-induced cognitive impairment [10]. On the other hand, benzodiazepines, which are primarily prescribed for anxiety and insomnia, have been implicated in inducing cognitive deficits such as declined reaction time, impaired attention, and anterograde amnesia that may persist beyond the duration of active drug use, and these agents also possess a strong potential to induce dementia in elderly patients [11]. Nevertheless, drug-induced cognitive impairment is often underestimated because cognitive impairment may result from either medication use or from the pathology of the disease itself. For instance, elderly patients with neurological disorders such as Parkinson’s disease or dementia may not be accurately assessed for drug-induced cognitive effects, as their cognitive deficits might be misattributed to the progression of their underlying condition rather than drug-induced ADEs [10,11]. This complexity emphasizes the importance of thorough evaluations through controlled trials of the etiologies involved with cognitive impairment. The Beers criteria has been utilized in clinical practices due to the substantial impact of these agents on cognitive function in the elderly patients, providing restrictions or reconsideration in their use, particularly among populations vulnerable to cognitive decline [9]. However, there are still high numbers of prescriptions for medications with high risk for cognitive impairment and decline in real clinical practice, predisposing patients to be at elevated risk for drug-induced cognitive impairment [12]. Moreover, our previous study demonstrated there is a 47% incidence of potentially inappropriate medication use in dementia patients, with the most prescribed medications being benzodiazepines, anticholinergics, and zolpidem [13]. This study reported a strong correlation of polypharmacy and multiple comorbidities, including schizophrenia, mood disorders, and Parkinson’s disease, with an increased likelihood of prescribing potentially inappropriate medication [13]. Nonetheless, randomized controlled trials (RCTs) addressing the essential clinical issues regarding medication- or disease-induced cognitive impairment as well as the clinical impacts of prescribing potentially inappropriate medication for cognitive impairment is lacking. Additionally, evidence on the incidence and seriousness of drug-induced cognitive impairment utilizing real-world data (RWD) is currently unavailable, despite there being a high prevalence of inappropriate medication use in real clinical settings. Hence, this study aims to evaluate the prevalence and severity of drug-induced cognitive impairment, identify etiologic medications associated with high incidence of drug-induced cognitive impairment, and investigate clinical predictors that may increase hospitalization risks from serious drug-induced cognitive impairment utilizing the Korean Adverse Event Reporting System Database (KAERS DB), a nationwide spontaneous ADE reporting system constructed by the Korean Institute of Drug Safety and Risk Management (KIDS).

## 2. Materials and Methods

### 2.1. Data Source and Definition

This study is a cross-sectional study conducted in accordance with Strengthening the Reporting of Observational Studies in Epidemiology (STROBE) guidelines [14]. This study was designed to explore drug-induced cognitive impairment and to identify etiologic factors by utilizing spontaneously reported ADE records obtained from the Korea Adverse Event Reporting System Database (KAERS DB), a nationwide pharmacovigilance system constructed by the Korean Institute of Drug Safety and Risk Management (KIDS, Ministry of Food and Drug Safety). ADE cases reported from 1 January 2012 to 31 December 2021 were included in the study. All ADE cases reported to the system underwent causality assessment, which were further verified by the healthcare professionals appointed by the KIDS. Causality of all ADE cases were verified based on medical charts, scientific pharmacovigilance data received by the manufacturers, and interviews with patient and healthcare professionals to minimize biases [15]. ADE types were confirmed with Medicinal Dictionary for Regulatory Activities (MedDRA) terminology, an international standard medical terminology for pharmacovigilance investigations, and the ADE reports were further classified into system organ classes (SOCs). All ADE reports regarding drug-induced cognitive impairment with “certain”, “probable/likely”, and “possible” causalities in accordance with the World Health Organization-Uppsala Monitoring Centre (WHO-UMC) criteria were included in the analysis. Prespecified MedDRA terminologies for cognitive impairment were “cognitive impairment”, “cognitive disorder”, “cognitive disorder aggravated”, “cognitive disturbance”, “cognitive function abnormal”, “cognitive linguistic deficit”, “major neurocognitive disorder”, “mild neurocognitive disorder”, “vascular cognitive impairment”, “minor cognitive motor disorder”, and “cognitive deterioration”. Any irrelevant ADE cases or ADE cases with masked etiologic medications (MSK coded) were excluded from the analysis; an MSK code is assigned to medication products that are marketed by less than 2 pharmaceutical companies. The following information was extracted from the ADE cases: (1) demographics (age, sex, and medical history), (2) information related to medication administrations (active ingredients, route and time of administrations), and (3) ADE information (causality, ADE types, seriousness, reporter types, and occurrence date). Serious adverse events (SAEs) were identified based on the International Conference on Harmonization (ICH) E2D guidelines and included any ADE cases related to death, life-threatening conditions, hospitalization, or prolongation of existing hospitalizations, persistent or significant disability or incapacity, birth defects or congenital abnormalities, and other medically significant events. The research protocol was approved by the institutional review board (IRB) of Kyung Hee University (Seoul, Republic of Korea) (KHSIRB-23-124) and KIDS (Ministry of Food and Drug Safety) (KIDS KAERS DB 2212A0073).

### 2.2. Statistical Analysis

All statistical analyses were conducted with R (version 4.1.0) and SPSS Statistics 26.0 (IBM SPSS Statistics for Windows, Armonk, NY, USA). Patient demographics as well as ADE frequency were analyzed with descriptive statistics. Age was expressed as median (interquartile range, IQR) in accordance with the results of Kolmogorov–Smirnov normality test. Disproportionality analysis was performed to investigate the association of serious adverse events (SAEs) and medication classes, and the effect size was estimated with reporting odds ratio (RORs) with corresponding 95% confidence intervals (CIs) along with Mantel–Haenszel adjusted *p*-values. Medications with at least 3 reported ADE cases for both non-serious AEs and SAEs were included in the disproportionality analysis. Multiple logistic regression with enter method was applied to identify clinical predictors that may increase risk of hospitalizations secondary to serious drug-induced cognitive impairment, and clinical predictors involve age, sex, comorbidities, and number of concomitant medications, which were prespecified based on the clinical plausibility. Sensitivity analysis was performed for ADE cases reported by patient aged 50 years or older to evaluate the clinical predictors associated with serious drug-induced cognitive impairment in the elderly. The effect size of each predictor was estimated with odds ratio (OR) with 95% CI. Any *p*-values < 0.05 were considered statistically significant.

## 3. Results

### 3.1. Demographic Information

Among 9185 ADE cases obtained from KAERS DB, a total of 254 drug-induced cognitive impairment cases were included in the analysis based on the WHO-UMC causality assessment from January 2012 to December 2021. Data on patient demographics are summarized in Table 1. Majority of ADEs were non-serious ADEs (*n* = 220, 86.6%). Among 34 SAE cases, ADE-induced hospitalization was accounted for 26 cases (76.4%). More than 50% of drug-induced cognitive impairment cases were reported in women (*n* = 142). Majority of ADEs were reported in patients aged 50 years or older (*n* = 180, 79.8%), with the most ADE cases reported in 70 to 79 age group (*n* = 94, 42.9%), followed by 60 to 69 group (*n* = 38, 17.4%). The most common comorbidity associated with drug-induced cognitive impairment were cancer (*n* = 37, 14.6%), followed by neuropsychiatric disorders (*n* = 35, 13.8%), vascular disease (*n* = 29, 11.4%), and musculoskeletal disorders (*n* = 12, 4.7%). More than 57% of cases primarily reported ADEs as abnormal or declined cognitive function, followed by perception-related impairment (*n* = 67, 26.4%), and social function and emotion-related impairment (*n* = 15, 5.9%).

### 3.2. Etiologic Medications for Drug-Induced Cognitive Imparirment ADEs

The most drug-induced cognitive impairment cases were reported with analgesics (*n* = 37, 14.6%), especially with morphine (*n* = 8, 0.8%) and tramadol (*n* = 8, 0.8%), followed by acetaminophen (*n* = 5, 2.0%) (Table 2). Zolpidem was responsible for 33 cases (13.0%) of drug-induced cognitive impairment. The prevalence of serious drug-induced cognitive impairment was 13.4%. Among 26 SAE cases related to ADE-induced hospitalizations, 15 cases were associated with anticancer agents, including fluorouracil and irinotecan (Table 3). Additionally, rasagiline and metoclopramide were accounted for four (15.4%) and three (11.5%) hospitalizations from drug-induced cognitive impairment, respectively.

### 3.3. Association between Medication Class and Serious Drug-Induced Cognitive Imparimrent

**Figure 1:**
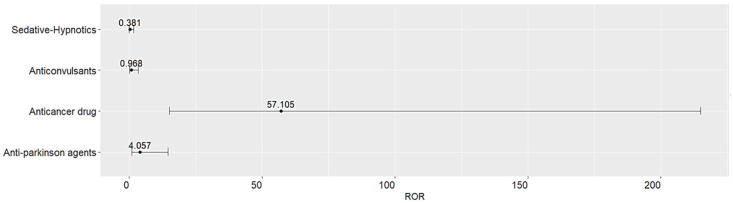
Association between serious cognitive impairment and medication class.

A significant association between the seriousness of drug-induced cognitive impairment and medication classes, including anticancer agents (ROR 57.105, 95% CI 15.174–215.909) and anti-Parkinson agents (ROR 4.057, 95% CI 1.121–14.688) was observed from the disproportionality analysis (Figure 1).

### 3.4. Clinical Predictors Increasing Risk of Hospitalization from Serious Drug-Induced Cognitive Impairment

**Figure 2:**
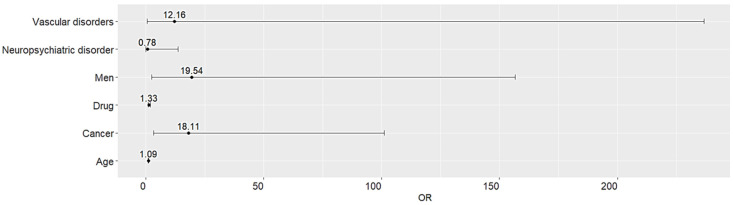
Clinical predictors increasing risk of hospitalization due to serious drug-induced cognitive impairment.

The clinical predictors associated with a substantially increased risk of serious drug-induced cognitive impairment include male sex (OR 19.540, 95% CI 2.440–156.647, *p* = 0.005) and cancer comorbidity (OR 18.115, 95% CI 3.246–101.101, *p* < 0.01) (Figure 2). Although statistically insignificant, the risk of hospitalization due to serious drug-induced cognitive impairment may increase with age, number of concomitant medications, and presence of vascular disorders.

### 3.5. Sensitivity Analysis

The clinical predictors associated with a substantially increased risk of serious drug-induced cognitive impairment in patients aged 50 years or older include male sex (OR 22.613, 95% CI 3.717–13.570, *p* < 0.001) and cancer comorbidity (OR 11.543, 95% CI 2.560–52.110, *p =* 0.001) (Table 4).

## 4. Discussion

Most drug-induced cognitive impairment cases were observed with analgesics, followed by sedative-hypnotics, anticonvulsants, and antidepressants. The prevalence of serious drug-induced cognitive impairment was 13.4%, and the majority of SAEs resulted in hospitalization or prolonged hospitalizations. Disproportionality analysis demonstrated a strong association between certain medication classes including anti-cancer drugs and anti-Parkinson agents, and a higher likelihood to report serious drug-induced cognitive impairment. In contrast, sedatives and anticonvulsants demonstrated an insignificant risk of reporting SAEs. Clinical predictors involved with increased risk of hospitalization due to drug-induced cognitive impairment include male sex and cancer comorbidities.

Cognitive impairment encompasses a wide spectrum of deficits involving thinking, learning, memory, judgment, and decision making [16]. Mild cognitive impairment (MCI) is referred to as the clinical stage between expected decline in cognitive function with aging and dementia, and is often considered as symptomatic predementia [17]. The risk of cognitive impairment generally increases with aging, with an estimated prevalence of 10 to 20% [8,17]. Moreover, multiple comorbidities and medication use predisposes elderly patients to increased cognitive impairment risks [8]. As suggested by the previous studies, this study revealed that clinical predictors involving patient demographics, etiologic medications, comorbidities, and number of concomitant medications play as contributing factors for drug-induced cognitive impairment.

Drug-induced cognitive impairment is often underestimated because cognitive impairment may result from either medication use or from the pathology of the disease itself. Parkinson’s disease (PD) is an age-related neurological disorder, and cognitive decline is considered one of the most common nonmotor symptoms [18]. A previous study revealed faster declines in various cognitive domains, particularly in executive function, memory, attention, and visuospatial areas, resulting in a higher cumulative dementia risk in patients diagnosed with PD [18]. PD itself induces cognitive impairment via primary pathogenic mechanism, including the reduction in dopaminergic cells and dopaminergic action in the substantia nigra, as well as changes in subcortical brain structures, which are closely correlated to cognitive decline in these patients [19]. On the other hand, this study suggested that anti-Parkinson agents including carbidopa-levodopa, rasagiline, and ropinirole were more likely to report serious drug-induced cognitive impairment in PD patients. Benztropine, an anticholinergic agent commonly prescribed to manage tremors in PD patients, is one of the most etiologic agents for cognitive impairment. As a result, benztropine is currently listed on the Beers criteria, and evidence suggests deficits in free recall, time perception, memory, and interference with storage of new information associated with benztropine [9,20]. In this study, rasagiline was responsible for all serious drug-induced cognitive impairment. Rasagiline, an irreversible monoamine oxidase B (MAO-B) inhibitor, increases dopamine concentration in the brain, thereby improving motor-related symptoms in PD [21]. Additionally, a randomized placebo-controlled study demonstrated the potential cognitive benefits of rasagiline on attention and executive function in PD patients without dementia, as dopamine is critical in controlling cognitive function [21]. However, another study suggested that rasagiline may not improve cognitive function in PD patients with MCI, implying variability in the cognitive effects of rasagiline depending on the stage of cognitive impairment [22]. Hence, further research is required to fully understand the impact of anti-Parkinson agents on cognitive function in PD patients and to identify optimal treatment strategies that balance motor symptom management with cognitive preservation. Comprehensive assessment and monitoring of cognitive function should be integrated into the routine care of PD patients to promptly identify and address any drug-induced cognitive impairment.

Interestingly, this study displayed a substantial association between anticancer agents and increased likelihood of reporting SAEs, and the majority of drug-induced cognitive impairment cases were reported by fluorouracil and irinotecan. Anticancer agents usually interfere with normal cellular functions, including transcriptions, apoptosis, and DNA repair [23]. Both irinotecan and 5-fluorouracil engage in either DNA repair or DNA synthesis, and these agents may induce cell death in specific areas in the brain, inducing biochemical and structural changes [23,24]. Until now, the evidence has been too limited to determine the causality of cognitive impairment in cancer patients [24]. Obviously, brain tumors may induce cancer-related cognitive impairments based on the specific lesions of the tumor location, and approximately 90% of patients with brain metastases show cognitive impairments correlated with total lesion volumes prior to chemotherapy [24]. Additionally, cognitive impairments, manifested in the slow speed of picture recognition and delayed word recall, were reported in patients with newly diagnosed large or locally advanced breast cancer. On the other hand, studies have suggested that the estimated prevalence of cognitive impairment is 13% to 70% in patients on chemotherapy, and a recent cohort indicated that the risk of chemotherapy-induced cognitive impairment is more prevalent in the elderly [23]. However, an evident mechanism of non-central nervous system cancer-mediated cognitive decline as well as chemotherapy-induced cognitive impairment is yet to be revealed. Hence, further research is warranted to elucidate the mechanism underlying cognitive impairment in cancer patients, thereby mitigating cognitive decline in this population.

Among 26 hospitalization cases from drug-induced cognitive impairment, 3 cases (11.5%) were induced by metoclopramide. Considering that only four cases of metoclopramide-induced cognitive impairment were reported from January 2012 to December 2021 to KAERS DB, the likelihood of reporting serious drug-induced cognitive impairment is considerably higher with metoclopramide, with an estimated ROR of 21.194. Metoclopramide functions as a dopamine receptor antagonist, serotonin 5-HT4 receptor agonist, and a weak 5-HT3 receptor antagonist, and is prescribed for the prevention of nausea and vomiting. Due to its antagonistic activity towards dopamine receptors, metoclopramide induce Parkinson’s disease-like symptoms with prolonged use [25]. However, evidence on metoclopramide-induced cognitive impairment is still lacking. Hence, further research is warranted to investigate the mechanism as well as the clinical impact of metoclopramide on cognitive impairment, especially those with predisposing factors such as advanced age or existing cognitive impairments.

On the contrary to previous studies, this study demonstrated higher risk of hospitalization from drug-induced cognitive impairment in men. Conventionally, women are at higher risk of cognitive impairment, as implied by a higher prevalence of dementia and cognitive dysfunction episodes [26]. However, this study demonstrated that men are more likely to develop severe drug-induced cognitive impairment that require hospitalization, and this could have attributed to differences in the comorbidity. In this study, anti-Parkinson agents were associated with high incidence of reporting SAEs. Usually, men are at two times higher risk of developing PD than women, and studies have suggested that men with PD generally exhibit poor cognitive abilities than women, particularly on frontal executive functions including attention and working memory, implying men are more susceptible to develop serious drug-induced cognitive impairment [27]. Moreover, men, particularly in the age group of 55 to 74, have a higher incidence rate of colorectal cancer (CRC), which are mainly treated with chemotherapy regimen with high risk of reporting SAES: 5-fluorouracil and irinotecan [28].

Although this study was not able to detect significantly increased SAE risk with aging and increased number of concomitant medications, these factors are the major risk factors for cognitive impairment [7]. Elderly populations are more susceptible to drug-induced cognitive impairment due to changes in pharmacokinetic/pharmacodynamic parameters as well as well as an increased number of concomitant medications secondary to multimorbidity, including cardiovascular/cerebrovascular disorders, cancer, and neuropsychiatric disorders [7]. Moreover, physiological changes, including brain volume, may predispose these patients to be at elevated risk for cognitive impairment [7]. Hence, further study is required to elucidate the complex interaction between aging, comorbidities, medication use, and cognitive impairment, especially in the elderly.

**Figure A1:**
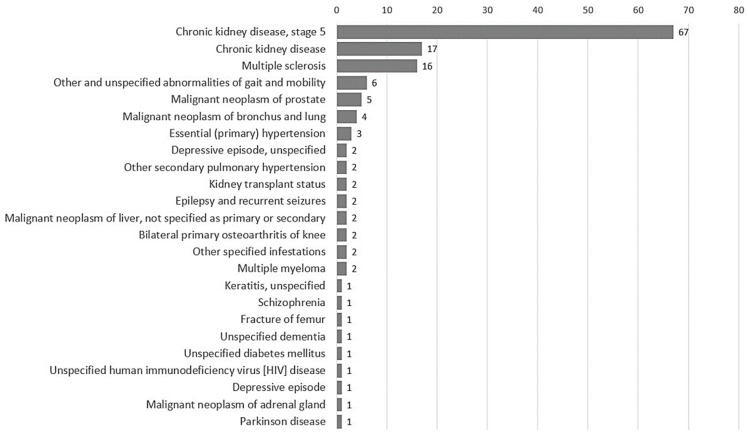
Patient comorbidities reported with MSK-coded ADEs.

In this study, we excluded MSK coded-ADE cases as we could not identify the etiologic agents. However, we need to acknowledge that the majority of patients who reported MSK coded-ADE cases had chronic kidney disease (CKD) as a major comorbidity, particularly end-stage renal disease (Appendix A Figure A1). Evidence has suggested that the risk of cognitive impairment is considerably higher in patients with declined renal function, accounting for an incidence of 10 to 40% [29]. CKD has multifaceted pathological features, including cerebrovascular and vascular diseases that contribute to the development of cognitive impairment [29]. Moreover, CKD patients administer numerous medications that may induce drug-interactions [29]. Nonetheless, despite a substantially high risk of cognitive impairment, concern on drug-induced cognitive impairment is underestimated when managing CKD patients. Thus, further studies as well as guidelines should be established for prescribing medications to CKD patients to minimize the risk of drug-induced cognitive impairment and optimize patient care.

The occurrence of drug-induced cognitive impairment can be easily unnoticed because of multiple underlying etiologies of cognitive impairment, such as comorbidities and medications. Healthcare providers usually pay closer attention when they prescribe or administer medications that are listed on the Beers criteria, including anticholinergics, benzodiazepines, and hypnotics. However, the majority of the time, they tend to pay less attention to the comorbidities of the patients. This study suggested that comorbidities such as PD, cancer, and CKD may also predispose patients to have an elevated risk of drug-induced cognitive impairment. Hence, there is a need for increased awareness among healthcare providers regarding the potential role of comorbidities in contributing to drug-induced cognitive impairment to improve patient safety and outcomes. Furthermore, guidance on the optimal use of medications beyond those listed on the Beers criteria in patients with high risk of developing drug-induced cognitive impairment should be established.

This study has several limitations. First, a cautious interpretation of the study results is necessary. As KAERS DB remains a spontaneous voluntary ADE reporting system, potential bias may arise from underreporting or selective reporting. Although the majority of ADE cases included in the study were reported by healthcare professionals, reporting bias may arise due to varying levels of interest and motivation among healthcare professionals on sharing the clinical impact of drug-induced cognitive impairment. Moreover, as we discussed previously, drug-induced cognitive impairment is less likely to be noticed or reported in the real world due to multiple etiologies contributing to cognitive impairment, particularly comorbidities, and the patients themselves may not recognize the symptoms or signs related to drug-induced cognitive impairment in their daily routine, which may result in small number of ADE cases pertaining to drug-induced cognitive impairment despite long follow-up durations. These factors may contribute to potentially incomplete or skewed data, which may not be generalized to determine apparent causality of medications with potential drug-induced cognitive impairment. Furthermore, being a spontaneous pharmacovigilance system, demographic information such as age, comorbidity, and concomitant medication were limited, which may have resulted in insignificant impacts of aging and the number of concomitant drug use as they pertain to the risk of serious drug-induced cognitive impairments and wider CIs. Additionally, this study did not provide sufficient evidence on the clinical significance of drug–drug interaction on the risk of drug-induced cognitive impairment and may have potential bias towards sex. Furthermore, this study was not able to determine specific cognitive domains such as learning, memory, perception, and problem solving affected by the medications. Different medications may exert varying impacts on cognitive process, and identifying the specific domains being affected can provide deeper insights into the potential risks associated with the medications. Hence, further research incorporating comprehensive cognitive assessments that specifically investigate drug effects on specific cognitive domains are warranted to enhance the understanding on drug-induced cognitive impairment and to provide optimal care for patients. Nonetheless, this study possesses clinical significance because it provides real-world evidence on the drug-induced cognitive impairment, subsequently promoting further research and a heighted awareness among healthcare providers. Nevertheless, large-scaled pharmacovigilance investigations on drug-induced cognitive impairment as well as risk stratifications on comorbidities and types of medications are highly warranted to optimize patient care.

## 5. Conclusions

In conclusion, the most etiologic medication classes for drug-induced cognitive impairment cases reported to KAERS DB were analgesics, followed by sedative-hypnotics. However, anticancer and anti-Parkinson agents were more likely to report serious drug-induced cognitive impairments. Male sex and cancer diagnoses are the major clinical predictors for increased risk of hospitalizations due to serious drug-induced cognitive impairment. However, further large-scaled studies are required because of the potential underreporting of drug-induced cognitive impairments in real practice settings, which is further complicated by the complexity of multiple contributing factors, such as comorbidities. Moreover, missing comprehensive demographic information within the database may have limited the detection of the clinical significance of age and number of concomitant administrations on the risk of serious drug-induced cognitive impairment. Despite these limitations, this study provides valuable real-world evidence on drug-induced cognitive impairment.

## Figures and Tables

**Table 1:** Demographic information.

Sex ^a^	
Men	107 (42.1%)
Women	142 (55.9%)
**Age ^b^ (65.1, IQR 20.0)**	
<10	1 (0.4%)
10 to 19	3 (1.2%)
20 to 29	12 (4.7%)
30 to 39	3 (1.2%)
40 to 49	11 (4.3%)
50 to 59	32 (12.6%)
60 to 69	38 (15.0%)
70 to 79	94 (37.0%)
≥80	16 (1.6%)
**Causality**	
Certain	19 (7.5%)
Probable/Likely	80 (31.5%)
Possible	155 (61.0%)
**Number of Concurrent Medications**	
1	235 (92.5%)
2	17 (6.7%)
3	1 (0.4%)
4	1 (0.4%)
**Comorbidities ^c^**	
Cancer	37 (14.6%)
Neuropsychiatric disorders	35 (13.8%)
Vascular disease	29 (11.4%)
Musculoskeletal disorders	12 (4.7%)
Diabetes	11 (4.3%)
Respiratory infection	3 (1.2%)
Others	48 (18.9%)
**ADE types**	
Non-SAE	220 (86.6%)
SAE	34 (13.4%)
**Report types ^d^**	
Doctors	96 (37.8%)
Pharmacists	77 (30.3%)
Nurses	64 (25.2%)
Others	11 (4.3%)
Types of drug-induced cognitive impairments	
Cognitive function declined or disorder	147 (57.9%)
Perception-related impairment	67 (26.4%)
Social function and emotion-related impairment	15 (5.9%)
Movement and problem-solving-related impairment	14 (5.5%)
Memory-related impairment	6 (2.4%)
Speech-related impairment	5 (2.0%)

^a^ Missing in 5 (2.0%) cases; ^b^ Missing in 44 (17.3%) cases; ^c^ Missing in 79 (31.1%) cases; ^d^ Missing in 6 (2.4%) cases. Abbreviation: IQR—interquartile range.

**Table 2:** Etiologic medications for drug-induced cognitive impairment.

Drug Class	No SAE (*n* = 220)	SAE (*n* = 34)	Total (*n* = 254)
**Analgesics**	**36 (16.4%)**	**0 (0.0%)**	**37 (14.6%)**
Acetaminophen	5 (2.3%)	0 (0.0%)	5 (2.0%)
Aspirin	4 (1.8%)	0 (0.0%)	4 (1.6%)
Celecoxib	2 (0.9%)	0 (0.0%)	2 (0.8%)
Codeine	2 (0.9%)	0 (0.0%)	2 (0.8%)
Dihydrocodeine	1 (0.5%)	0 (0.0%)	1 (0.4%)
Loxoprofen	2 (0.9%)	0 (0.0%)	2 (0.8%)
Meloxicam	2 (0.9%)	0 (0.0%)	2 (0.8%)
Morphine	8 (3.6%)	0 (0.0%)	8 (0.8%)
Naproxen sodium	1 (0.5%)	0 (0.0%)	1 (0.4%)
Oxycodone	1 (0.5%)	0 (0.0%)	1 (0.4%)
Tramadol	8 (3.6%)	0 (0.0%)	8 (0.8%)
**Sedative-Hypnotics**	**31 (14.1%)**	**2 (5.9%)**	**33 (13.0%)**
Zolpidem	31 (14.1%)	2 (5.9%)	33 (13.0%)
**Antidepressants**	**18 (%)**	**0 (0.0%)**	**18 (7.1%)**
Amitriptyline	6 (2.7%)	0 (0.0%)	6 (2.4%)
Duloxetine	1 (0.5%)	0 (0.0%)	1 (0.4%)
Escitalopram	5 (2.3%)	0 (0.0%)	5 (2.0%)
Fluoxetine	2 (0.9%)	0 (0.0%)	2 (0.8%)
Tianeptine	2 (0.9%)	0 (0.0%)	2 (0.8%)
Venlafaxine	2 (0.9%)	0 (0.0%)	2 (0.8%)
**Anticonvulsants**	**20 (9.09%)**	**3 (8.8%)**	**23 (9.06%)**
Divalproex	2 (0.9%)	0 (0.0%)	2 (0.8%)
Oxcarbazepine	1 (0.5%)	0 (0.0%)	1 (0.4%)
Pregabalin	4 (1.8%)	0 (0.0%)	4 (0.4%)
Gabapentin	8 (3.6%)	0 (0.0%)	8 (0.8%)
Sodium valproate	3 (1.4%)	0 (0.0%)	3 (1.2%)
Topiramate	10 (4.5%)	3 (8.8%)	13 (5.1%)
**Anticancer drugs**	**3 (1.4%)**	**15 (44.12%)**	**18 (7.09%)**
Fluorouracil	0 (0.0%)	10 (29.4%)	10 (4.0%)
Irinotecan	0 (0.0%)	5 (14.7%)	5 (2.0%)
Megestrol	1 (0.5%)	0 (0.0%)	1 (0.4%)
Methotrexate	1 (0.5%)	0 (0.0%)	1 (0.4%)
Paclitaxel	1 (0.5%)	0 (0.0%)	1 (0.4%)
**Anti-cholesterol Drug**	**13 (5.9%)**	**0 (0.0%)**	**13 (5.1%)**
Ezetimibe	6 (2.7%)	0 (0.0%)	6 (2.4%)
Rosuvastatin	7 (3.2%)	0 (0.0%)	7 (2.8%)
**Anticholinergic**	**12 (5.5%)**	**1 (2.9%)**	**13 (5.1%)**
Benztropine	11 (5.0%)	1 (2.9%)	12 (4.7%)
Glycopyrrolate	1 (0.5%)	0 (0.0%)	1 (0.4%)
**Antihistamine**	**10 (4.5%)**	**0 (0.0%)**	**10 (3.9%)**
Chlorpheniramine	3 (1.4%)	0 (0.0%)	3 (1.2%)
Bepotastine	1 (0.5%)	0 (0.0%)	1 (0.4%)
Dimenhydrinate	2 (0.9%)	0 (0.0%)	2 (0.8%)
Hydroxyzine	1 (0.5%)	0 (0.0%)	1 (0.4%)
Mequitazine	2 (0.9%)	0 (0.0%)	2 (0.8%)
Triprolidine	1 (0.5%)	0 (0.0%)	1 (0.4%)
**Anti-Parkinson agents**	**7 (3.2%)**	**4 (11.8%)**	**11 (4.3%)**
Carbidopa-levodopa	6 (2.7%)	0 (0.0%)	6 (2.4%)
Rasagiline	0 (0.0%)	4 (11.8%)	4 (1.6%)
Ropinirole	1 (0.5%)	0 (0.0%)	1 (0.4%)
**Anxiolytic**	**14 (3.6%)**	**0 (0.0%)**	**14 (5.5%)**
Alprazolam	6 (2.7%)	0 (0.0%)	6 (2.4%)
Buspirone	2 (0.9%)	0 (0.0%)	2 (0.8%)
Clonazepam	2 (0.9%)	0 (0.0%)	2 (0.8%)
Diazepam	1 (0.5%)	0 (0.0%)	1 (0.4%)
Lorazepam	1 (0.5%)	0 (0.0%)	1 (0.4%)
Midazolam	2 (0.9%)	0 (0.0%)	2 (0.8%)
**Antipsychotic**	**6 (2.7%)**	**1 (2.94%)**	**7 (2.8%)**
Clozapine	0 (0.0%)	1 (2.94%)	1 (0.4%)
Olanzapine	1(0.5%)	0 (0.0%)	1 (0.4%)
Quetiapine	5 (2.3%)	0 (0.0%)	5 (2.0%)
**Dementia drugs**	**2 (0.9%)**	**0 (0%)**	**2 (0.8%)**
Donepezil	1 (0.5%)	0 (0.0%)	1 (0.4%)
Memantine	1 (0.5%)	0 (0.0%)	1 (0.4%)
**Others**	**48 (21.8%)**	**8 (23.5%)**	**56 (22.0%)**

**Table 3:** Etiologic agents associated with hospitalizations.

Medications	Cases (*n* = 26)
Topiramate	1 (3.8%)
Clozapine	1 (3.8%)
Metoclopramide	3 (11.5%)
Rasagiline	4 (15.4%)
Calcium folinate	5 (19.2%)
Irinotecan	5 (19.2%)
Fluorouracil	5 (19.2%)
Methylprednisolone	2 (7.7%)

**Table 4:** Clinical predictors associated with increased risk of serious drug-induced hospitalization.

Clinical Predictors	OR (95% CI)	*p*-Value
**Age**	0.998 (0.908–1.100)	0.967
**Men**	22.613 (3.717–137.570)	<0.001
**Drug**	1.342 (0.939–1.920)	0.106
**Neuropsychiatric disorder**	0.495 (0.034–7.17)	0.606
**Vascular disorders**	2.911 (0.160–53.050)	0.471
**Cancer**	11.543 (2.560–52.110)	0.001

## Data Availability

The data underlying this article cannot be shared publicly due to the ethical and privacy policies of the Korean Institute of Drug Safety & Risk Management (Ministry of Food and Drug Safety). The data will be shared upon reasonable request to the corresponding author.
